# Computed tomography-based structural rigidity analysis can assess tumor- and treatment-induced changes in rat bones with metastatic lesions

**DOI:** 10.1186/s12885-024-12374-8

**Published:** 2024-06-26

**Authors:** Michael S. Bohanske, Kaveh Momenzadeh, Peer van der Zwaal, Frederik J.H. Hoogwater, Esther Cory, Peter Biggane, Brian D. Snyder, Ara Nazarian

**Affiliations:** 1 Musculoskeletal Translational Innovation Initiative, Beth Israel Deaconess Medical Center, Harvard Medical School, 330 Brookline Avenue, RN123, Boston, MA 02215 USA; 2 Department of Emergency Medicine, Maine Medical Center, Tufts University School of Medicine, Portland, ME USA; 3 Department of Orthopedic Surgery & Trauma Unit, Haaglanden Medical Center, The Hague, The Netherlands; 4 Department of Surgery, University Medical Center Groningen, Groningen, The Netherlands; 5 Department of Orthopaedic Surgery, Boston Children’s Hospital, Harvard Medical School, Boston, MA USA; 6 Department of Orthopaedic Surgery, Yerevan State Medical University, Yerevan, Armenia; 7 Department of Mechanical Engineering, Boston University, Boston, MA USA

**Keywords:** Breast cancer, Metastasis, Structural rigidity, Osteolytic lesions, CT-based structural rigidity analysis, CTRA, DEXA, Fracture risk, Clinical decision support tool, CDS tool

## Abstract

**Background:**

Breast cancer (BrCa) is a predominant malignancy, with metastasis occurring in one in eight patients, nearly half of which target the bone, leading to serious complications such as pain, fractures, and compromised mobility. Structural rigidity, crucial for bone strength, becomes compromised with osteolytic lesions, highlighting the vulnerability and increased fracture risk in affected areas. Historically, two-dimensional radiographs have been employed to predict these fracture risks; however, their limitations in capturing the three-dimensional structural and material changes in bone have raised concerns. Recent advances in CT-based Structural Rigidity Analysis (CTRA), offer a promising, more accurate non-invasive 3D approach. This study aims to assess the efficacy of CTRA in monitoring osteolytic lesions’ progression and response to therapy, suggesting its potential superiority over existing methodologies in guiding treatment strategies.

**Methods:**

Twenty-seven female nude rats underwent femoral intra-medullary inoculation with MDA-MB-231 human breast cancer cells or saline control. They were divided into Control, Cancer Control, Ibandronate, and Paclitaxel groups. Osteolytic progression was monitored weekly using biplanar radiography, quantitative computed tomography (QCT), and dual-energy X-ray absorptiometry (DEXA). CTRA was employed to predict fracture risk, normalized using the contralateral femur. Statistical analyses, including Kruskal-Wallis and ANOVA, assessed differences in outcomes among groups and over time.

**Results:**

Biplanar radiographs showed treatment benefits over time; however, only certain time-specific differences between the Control and other treatment groups were discernible. Notably, observer subjectivity in X-ray scoring became evident, with significant inter-operator variations. DEXA measurements for metaphyseal Bone Mineral Content (BMC) did not exhibit notable differences between groups. Although diaphyseal BMC highlighted some variance, it did not reveal significant differences between treatments at specific time points, suggesting a limited ability for DEXA to differentiate between treatment effects. In contrast, the CTRA consistently demonstrated variations across different treatments, effectively capturing bone rigidity changes over time, and the axial- (EA), bending- (EI), and torsional rigidity (GJ) outcomes from the CTRA method successfully distinguished differences among treatments at specific time points.

**Conclusion:**

Traditional approaches, such as biplanar radiographs and DEXA, have exhibited inherent limitations, notably observer bias and time-specific inefficacies. Our study accentuates the capability of CTRA in capturing real-time, progressive changes in bone structure, with the potential to predict fractures more accurately and provide a more objective analysis. Ultimately, this innovative approach may bridge the existing gap in clinical guidelines, ushering in enhanced Clinical Decision Support Tool (CDST) for both surgical and non-surgical treatments.

## Introduction

Cancer is a widespread and pervasive disease that affects populations worldwide [1]. According to estimates, the United States will experience 1.9 million new cancer diagnoses and 609,820 cancer-related deaths in 2023 [2]. Of these, more than 250,000 will be diagnosed with breast cancer (BrCa), and more than 42,000 will die from the disease. One in eight women who receive a breast cancer diagnosis will experience metastasis, with nearly 50% of these metastases manifesting in bone. Almost two-thirds of patients with bone metastases will experience a range of adverse effects, such as pain, compression of the nerve root or spinal cord, pathological fractures, paralysis, restricted mobility, infiltration of the bone marrow, and hypercalcemia of malignancy [3]. Studies have demonstrated that treatment protocols have made significant progress in extending the lifespan of patients with BrCa, enhancing their quality of life by reducing discomfort and improving physical mobility. To facilitate treatment modalities and assess disease progression following metastasis, it is imperative to conduct prompt and precise analysis of alterations in bone structure, particularly when considering surgical fixation.

Structural rigidity represents the ability of bone to resist deformation as a product of bone tissue strength and geometry [4, 5]. Both of these are critical elements that contribute to the bone’s structural rigidity [6] and must be considered when evaluating the extent to which an osteolytic lesion has influenced the mechanical behavior of the host bone. This lesion becomes the weakest cross-section of the bone, causing a reduction in load-bearing capacity (LBC) and increasing the risk of bone failure and fracture. In the past, physicians have utilized two-dimensional radiographs for predicting fracture risk secondary to the degree of osteolytic lesions. However, using this approach to determine fracture risk for a three-dimensional object, such as bone, only considers the 2D projection of the geometry and fails to evaluate the changes that have occurred to the 3D structure and the material properties of the bone [7]. Therefore, using two-dimensional X-rays to determine fracture risk may be inaccurate [8] and may lead to unnecessary surgical interventions that can further compromise patient health and recovery.

Rigidity, the structural property that measures the resistance of bone to deformation under axial compression, bending, or torsional loading, combines both the material and geometric properties of bone into a single variable [9, 10]. Axial, bending, and torsional rigidities, measured non-invasively on sequential trans-axial quantitative computed tomography (QCT) images throughout bone, may be used to identify progressive changes in bone structural properties. Fracture load and location can then be predicted by the cross-section calculated to have the minimum rigidity. Using composite beam theory and serial transaxial CT images, we can determine the structural rigidity of bone and, in turn, predict its fracture risk threshold. This noninvasive 3D approach to predicting whole bone fracture risk considers the location and geometry of the osteolytic lesions, the biological activity of the neoplasm, and the material properties of the bone. This technique called CT-based Structural Rigidity Analysis (CTRA), has demonstrated 100% sensitivity and 90% specificity in predicting fracture in human femurs with metastatic lesions [11]. Precise evaluation of structural rigidity has the potential to enhance the physicians’ ability to identify the likelihood of fractures and monitor the progress of treatment, thereby resulting in more effective prevention and treatment strategies [12].

Paclitaxel, a common treatment for breast cancer and other solid tumors, displays varying sensitivities among different tumor types [13, 14]. Notably, MDA-MB-231 cells in breast cancer cell lines reveal varying responses to this treatment. Some studies have suggested resistance or the development of tolerance to paclitaxel within a subset of MDA-MB-231 cells, emphasizing the importance of evaluating treatment responses in these patients. Bisphosphonates (BP), primarily recognized for their role in inhibiting osteoclasts and treating bone metastases in breast cancer patients, have gained attention due to their effects in reducing tumor burden within bones. In particular, the bisphosphonate ibandronate has been studied for its impact on MDA-231 human breast cancer cells in bone metastases, leading to a heightened rate of apoptosis in these cells [15].

The objective of this research is to evaluate the ability of CTRA to assess the progression of osteolytic lesions and their response to therapeutic interventions in a rat model of metastatic bone lesions. *We hypothesize that CTRA can evaluate tumor- and treatment-induced spatial and temporal changes in bone mechanics better than existing methodologies.* The potential outcome of this approach is an improved technique to aid clinicians in diagnosing fracture risk and evaluating the effectiveness of treatment, thereby developing a more effective treatment strategy.

## Materials and methods

Animal experimental protocols were approved by the Institutional Animal Care and Use Committee (IACUC) at Beth Israel Deaconess Medical Center. A total of 27 NIHRNU-M female nude rats (eight-week-old, mass: 100–150 g) were obtained from Charles River Laboratories (Charles River, Charlestown, MA, USA). In this study, we utilized a human breast carcinoma cell line, MDA-MB-231 (courtesy of Dr. Theresa Guise, MD Anderson Cancer Center), widely employed in nude murine models to investigate the biology of skeletal metastases [16–22]. We created reproducible, site-specific lesions by directly inoculating these cells into the femoral intra-medullary canal. This approach has the advantage of minimizing the number of metastatic tumors throughout the body, thereby reducing tumor burden and enabling animals to live with the disease for extended periods. Although the tumor is not genuinely metastatic, it provides a reliable model for breast cancer tumor activity within a bone environment, simulating osteolytic activity typically observed in metastatic cancers.

**Fig. 1:**
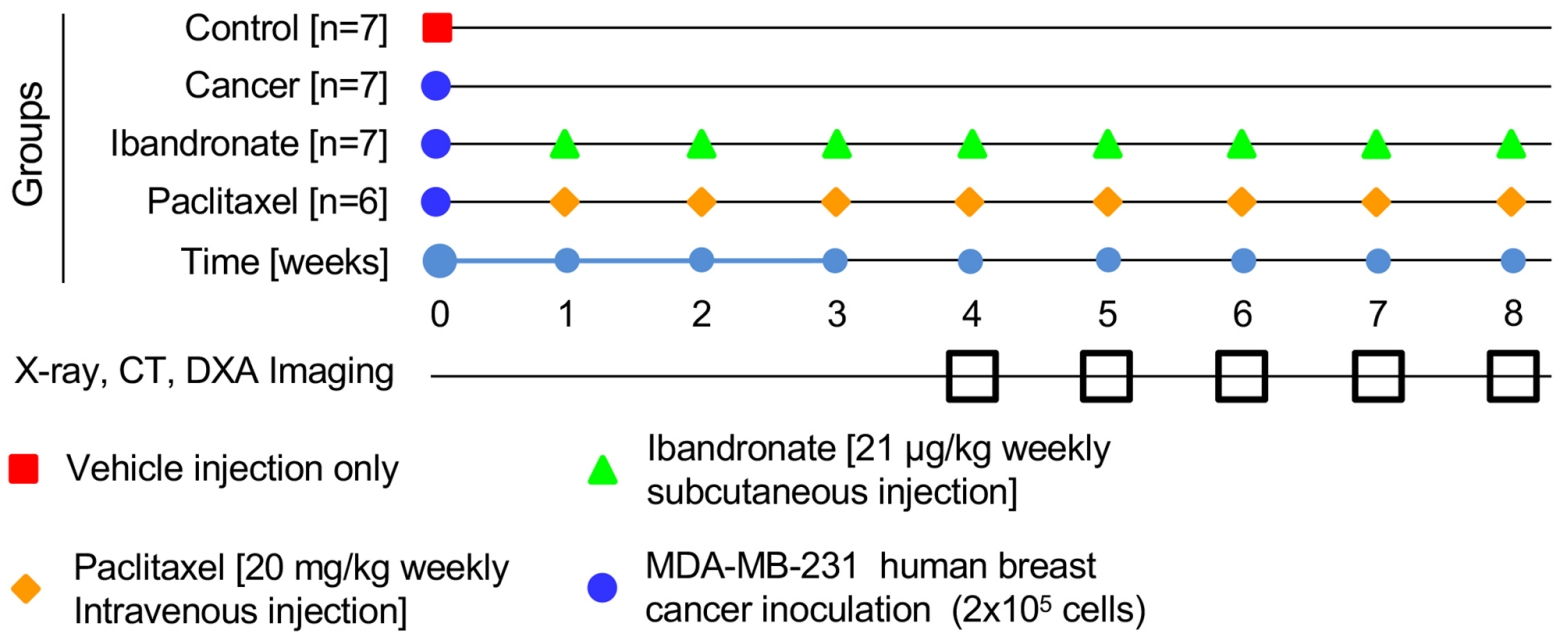
Provides a visual summary of the experimental timeline, depicting the schedule of various interventions and assessments for the different groups in the study. The red square indicates the period during which sham surgery was performed, marked as the starting point. The blue circle denotes the inoculation of the cancer group with MDA-MB-231 human breast cancer cells. Treatment intervals are illustrated by green triangles for Ibandronate and orange diamonds for Paclitaxel, signifying subcutaneous and intravenous administration, respectively. The hollow black boxes at the bottom of the timeline mark the weeks when X-ray, CT, and DEXA imaging were conducted to monitor changes and outcomes within the study

The control group (Control) comprised seven rats randomly assigned to undergo sham surgery in which no cancer cells were administered. The remaining cohort of 21 rats was selected to receive inoculations of MDA-MB-231 human breast cancer cells. These rats were subsequently divided into three groups: Cancer control (CA, consisting of 7 rats), Ibandronate treatment (IBAN, consisting of 7 rats), and Paclitaxel treatment (PAC, consisting of 7 rats, 1 death due to anesthesia, final group size of 6) [Fig. 1]. All 27 rats survived the study, and through subjective manual examination, it was determined that 2 rats from the CA group experienced destabilized fractures, which were identified by palpating the bones to assess their integrity. These fractures were subsequently followed by healing at the lesion site. Ibandronate was administered via subcutaneous injection at a dose of 21 µg/kg weekly. Paclitaxel was delivered weekly through intravenous injection at 20 mg/kg. The contralateral, non-surgical limb served as the internal control for each animal, eliminating biological variation between animals.

### Surgical preparation and treatment groups

The experimental design required a simulated metastatic lesion in a reproducible location. For this reason, a method of femoral intra-medullary inoculation was used to create site-specific lesions. The surgical procedure was executed by a surgeon in a sterile environment while adhering to aseptic conditions. The rats were subjected to anesthesia through induction inhalation of 5% isoflurane, followed by maintenance of 2% isoflurane via a nose cone. A small incision was made on the medial surface of the knee taking caution to cut through the skin only. Muscle tissue was then divided using the blunt tips of scissors to minimize bleeding and trauma until the medial condyle of the femur was visible. Under fluoroscopic guidance, a dental drill was used to create a portal through the cortex of the medial condyle at approximately a 45^o^ elevation from the shaft of the femur. A needle was then used to extend the portal into the medullary canal; the proper entrance was confirmed by visible bleeding within the portal and fluoroscopic images displaying the needle in the canal. MDA-MB-231 cancer cells (2 × 10^5^ cells suspended in 50 µL of 0.9% saline) were injected into the canal [23] using a 33 GA needle. Control animals received an equal volume injection of saline only. The portal was sealed with bone wax, and the incision was closed using surgical staples and coated with triple-antibiotic ointment.

Post-operative care included close observation of anesthetized animals placed under heat lamps to maintain body temperature until they regained full consciousness and the ability to move freely around the cages. All rats received buprenorphine hydrochloride (Bupranex, Reckitt Benckiser LLC, Parsippany, NJ, USA) at a dose of 0.3 mg/kg every 6 h for 48 h following surgery to mitigate any potential pain. Animals were early euthanized if they exhibited severe post-operative complications such as uncontrollable infections, significant surgical complications, or behavioral changes indicating distress, including lack of mobility, excessive weight loss of more than 20%, or inadequate pain management. The study concluded with the euthanasia of all remaining animals at the end of week 8.

### Radiological assessment and image analysis

Commencing from the fourth week, the animals were subjected to anesthesia, where both femurs were monitored weekly using biplanar radiography (HP Faxitron Cabinet X-ray system, Model 43,855 A, McMinnville, OR, USA), serial QCT scanning (XCT Research SA+, Stratec, Pforzheim, Germany), and dual-energy X-ray absorptiometry (DEXA, Lunar PIXImus2, GE Healthcare, Waukesha, Wisconsin, USA) from the distal metaphysis to the proximal diaphysis, to monitor the regression/progression of the resultant osteolytic lesions.

**Fig. 2:**
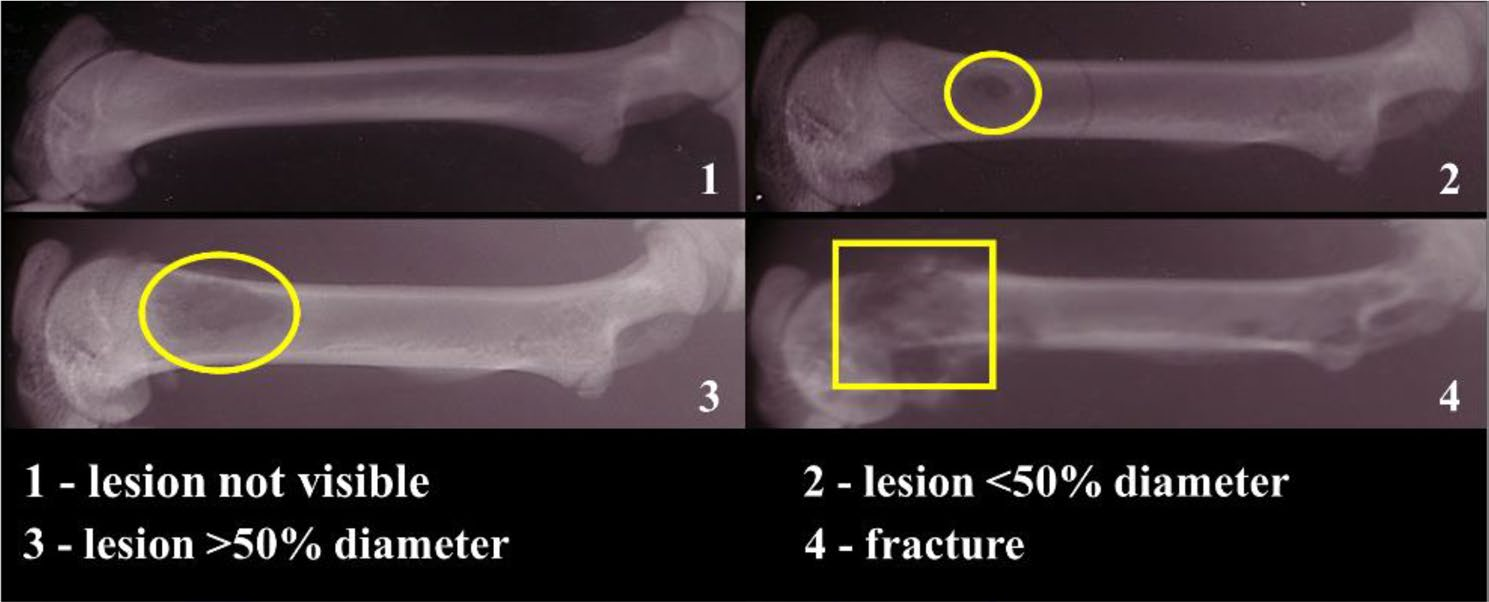
Illustration of the categorical scale for radiographic film analysis

Anterior-Posterior biplanar radiographs were blinded and analyzed similarly to current clinical guidelines based on lesion size by two independent observers using a four-point categorical scale: 1 = no lesion, 2 = lesion < 50% femur diameter, 3 = lesion > 50% femur diameter, 4 = fracture [Fig. 2]. All scores were based on the largest visible lesion. This scoring system was devised in correlation with current clinical criteria used to evaluate lesions that are considered at significant risk of fracture (those lesions > 50% the diameter of the site) [24–28]. The scores obtained from both observers were then averaged.

**Fig. 3:**
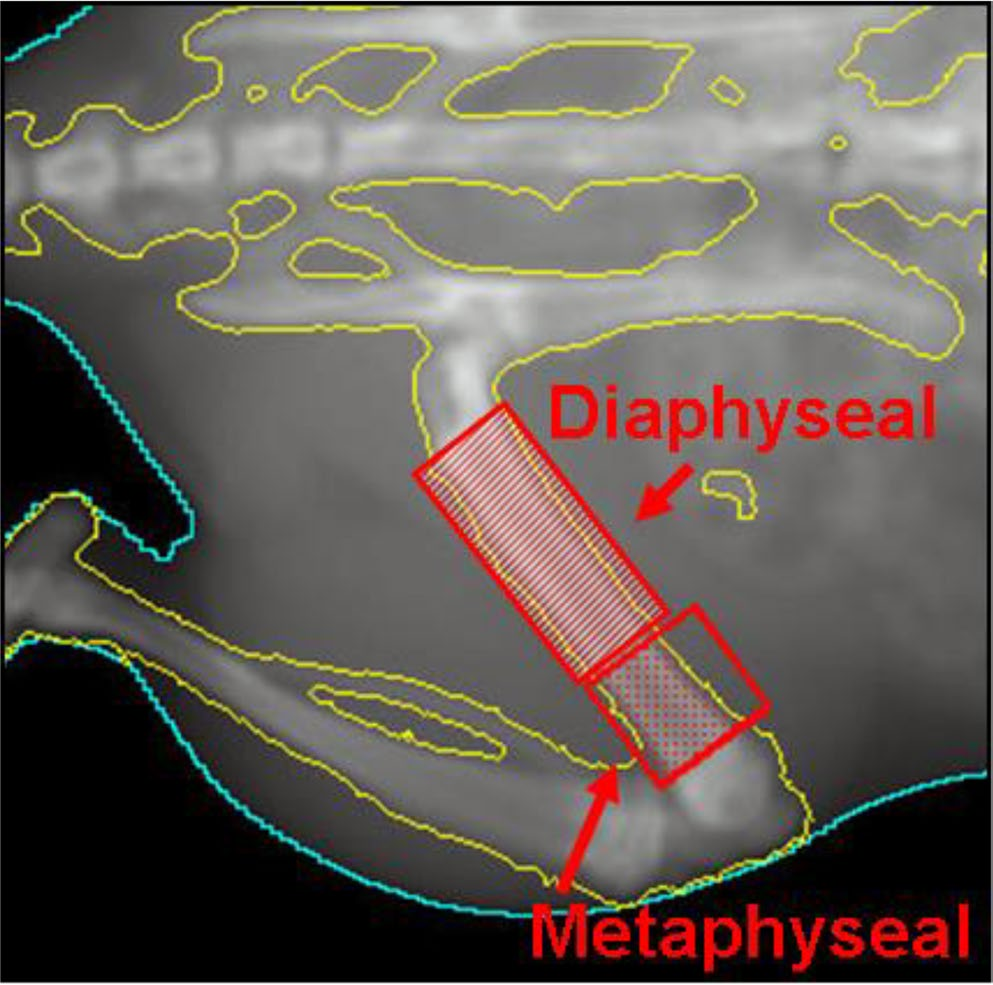
The diaphyseal (cortical) and metaphyseal (trabecular) regions of interest for DEXA BMC and BMD analysis, using the PIXImus software

The Bone Mineral Content (BMC) and Bone Mineral Density (BMD) at two distinct regions of interest, particularly the distal metaphyseal region (predominantly trabecular bone) and the mid-diaphyseal region (predominantly cortical bone), were calculated from the DEXA scans [Fig. 3]. A 10% reduction in BMC compared to the contralateral femur was used as a threshold for fracture. A 10% reduction in BMC as a threshold for fracture risk is consistent with findings by Ullom-Minnich et al., where it was associated with 2–3 times increase in the fracture risk [29].

**Fig. 4:**
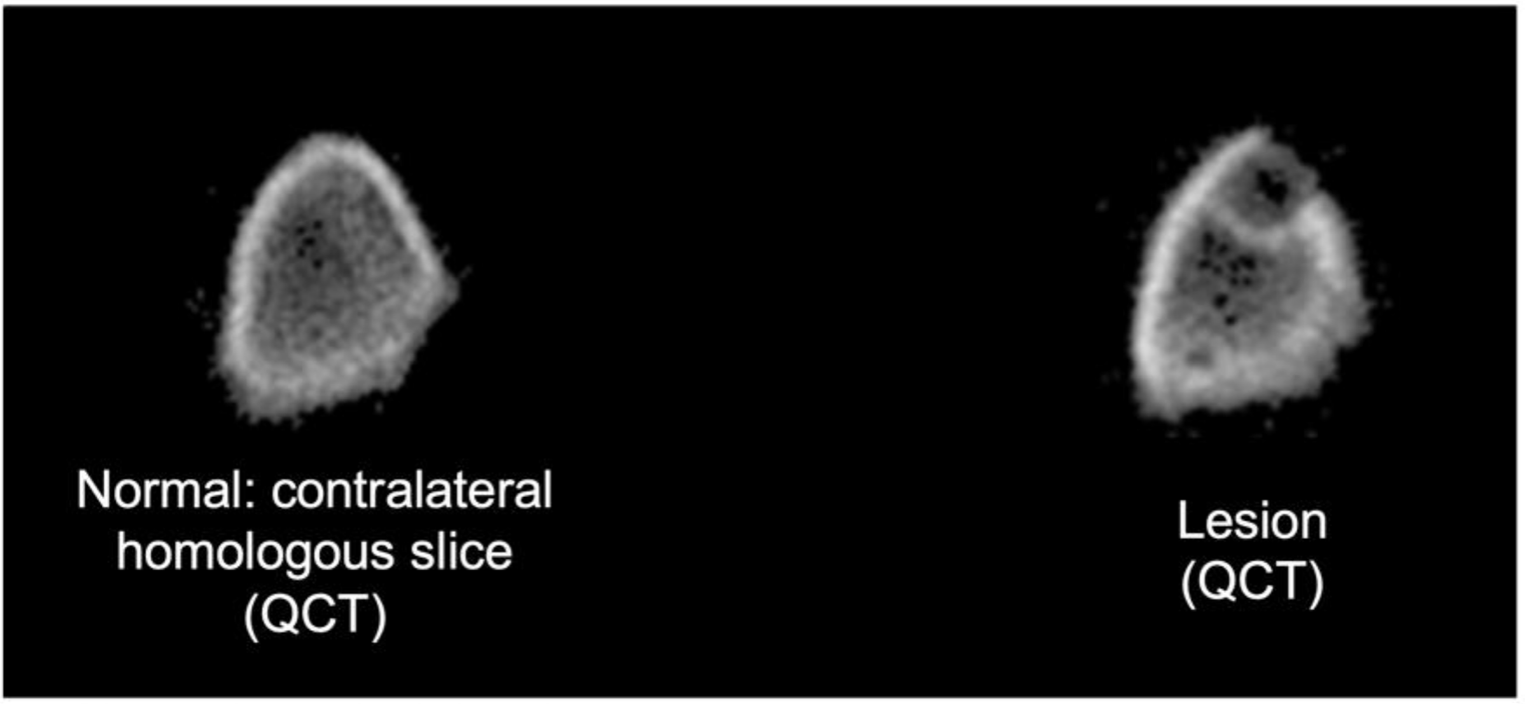
Homologous slices of right and left femora where the lytic lesion is visible. Rigidity values calculated from each slice were normalized by the non-surgical limb to account for biological variation and to serve as intra-animal control

The QCT Research SA + was used to obtain QCT images of the distal femora [Fig. 4]. The animals were restrained similarly to human anatomical positioning (simulated bipedal orientation of the femora) to create CT slices perpendicular to the femora. Serial/sequential trans-axial slices with 100 μm in-plane voxel size and 490 μm slice thickness were obtained, covering an area of approximately 15 mm proximal to the growth plate (metaphyseal and distal diaphyseal regions). QCT scanning was obtained starting week 4 (T_4_) until eight weeks post-inoculation (T_8_).

**Fig. 5:**
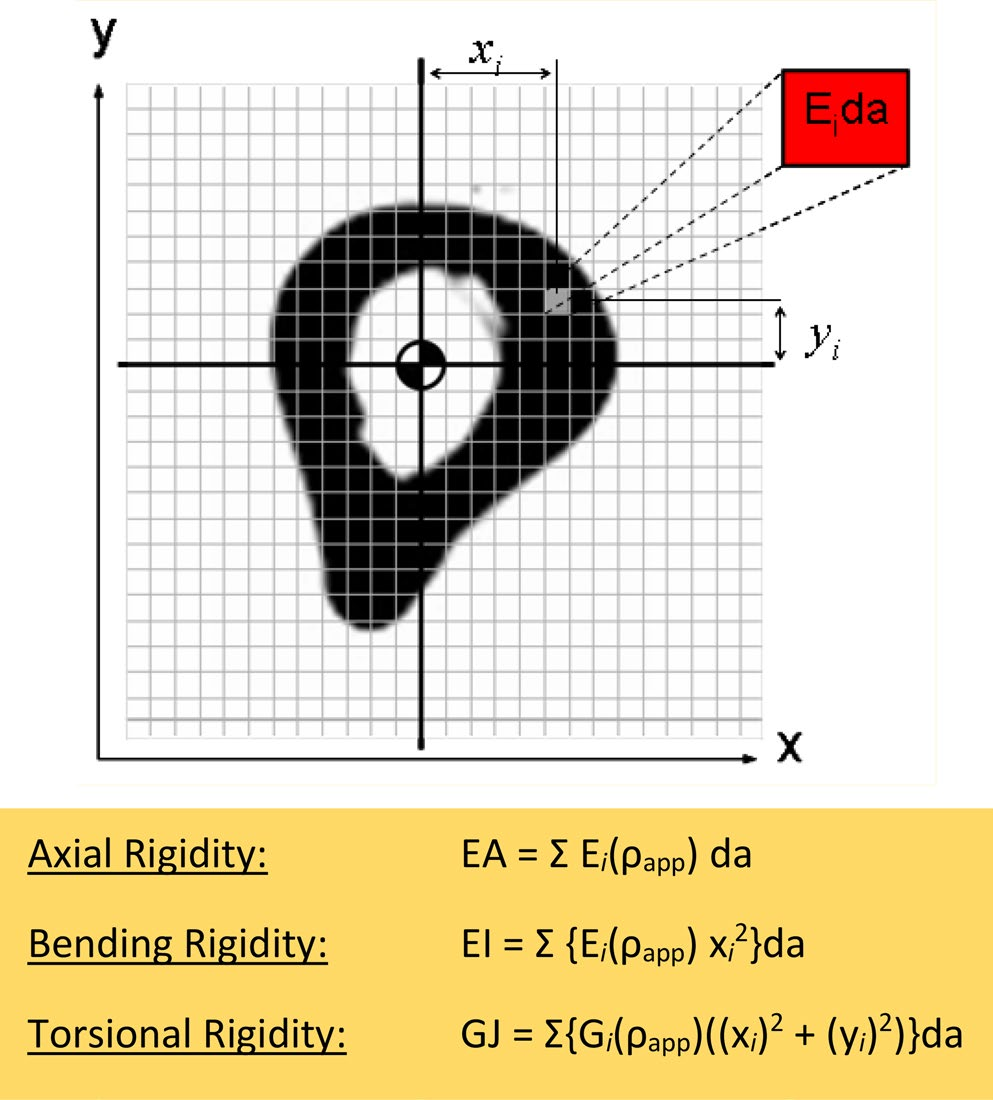
Modulus-weighted pixel is summation with its distance from the centroid in the calculations of EA, EI and GJ. Where E is the modulus of elasticity of pixel, i, with density, ρ_app_, da is the area of the pixel, xi and yi are the distances to the coordinates of the modulus-weighted centroid

Structural rigidity analysis is a technique to estimate fracture risk in bones [30], where the weakest cross-section of the bone is thought to govern fracture risk. In other words, it is assumed that overall bone strength is governed by the cross-section exhibiting the lowest structural rigidity indices [8, 31–34]. These indices are a product of the modulus of elasticity and minimum cross-sectional area (EA, axial rigidity); modulus of elasticity and moment of inertia (EI, bending rigidity); and shear modulus and polar moment of inertia (GJ, torsional rigidity). The QCT DICOM image files were analyzed using an in-house developed CTRA software package based on the ImageJ (NIH) system to calculate rigidities [35] [Fig. 5]. It is important to note that the rigidity indices—EA, EI, and GJ—are predictive markers, not direct fracture evidence.

Rigidity values were normalized by the homologous slice of the normal contralateral femur, presenting all data as a relative change from the contralateral bone. A reference value of 1.0 indicates equal rigidity value to that of the homologous slice. This normalization was performed to account for biological variation and to utilize the contralateral limb as intra-animal control. For each specimen, the cross-section with the minimum predicted rigidity is assumed to be the failure initiation site. The incidence of fracture was predicted when either EA, EI, or GJ index was reduced by 33% compared to the contralateral side (Δ ≥ 33%) [30].

### Statistical analysis

We assessed differences in the X-ray, CT-based rigidity analysis (CTRA), and BMC outcomes among the treatment groups and over time. We used the Kolmogorov- Smirnov test to assess whether data followed a normal (Gaussian) distribution. For X-ray observations, scores were analyzed using a non-parametric test suitable for ordinal data such as X-ray scores. The Kruskal-Wallis test detected significant differences in X-ray scores among the treatment groups at each time point. *Post-hoc* pairwise comparisons were conducted using Dunn’s test with a Bonferroni correction for multiple comparisons when a significant main effect was identified. For the CTRA and BMC measurements, a two-way mixed effects analysis of variance (ANOVA) was conducted to investigate the main effects of treatment and time, as well as their interaction. The model was fitted with treatment as the between-subjects factor and time as the within-subjects factor. When a significant main effect or interaction was identified, *post-hoc* pairwise comparisons were performed using Tukey’s HSD test to explore specific group differences. All statistical analyses were performed using GraphPad Prism (Version 10, GraphPad Software, San Diego, California, USA), and the significance level was set at *p* < 0.05. All data are presented as mean ± standard deviation.

## Results

**Fig. 6:**
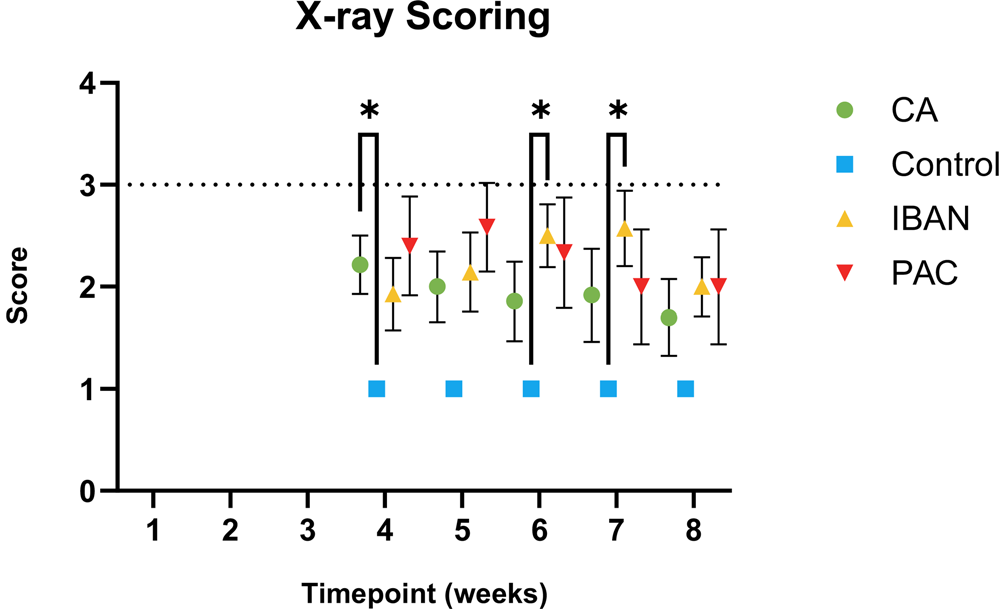
Average X-ray Scores. 1 = no lesion, 2 = lesion < 50% femur diameter, 3 = lesion > 50% femur diameter, 4 = fracture. The dotted line represents the fracture prediction threshold * Indicates P ≤ 0.05, ** indicates P ≤ 0.01, and *** indicates P ≤ 0.001

Biplanar radiographs demonstrated a beneficial effect of treatment over time (two-way mixed effects ANOVA, *p* = 0.025); however, *post-hoc* pairwise comparisons could only distinguish between Control and CA at week four and Control and IBAN at weeks 6 and 7 and did not distinguish any difference between the treatment groups (i.e., PAC and IBAN). Using a 4-point categorical scale, animals with a score exceeding two were deemed to be at risk of experiencing fractures. The X-ray scores for the control group (without any pathological lesion) remained consistent throughout the study. This discrepancy highlights the potential subjectivity and inherent observer bias that might be present in visual scoring assessments using X-rays. Additionally, Fig. 6 provides an average X-ray-scoring snapshot of the study by two independent observers. Inter-operator Kappa testing indicated significant differences between the two independent observers’ scores (*p* = 0.001).

**Fig. 7:**
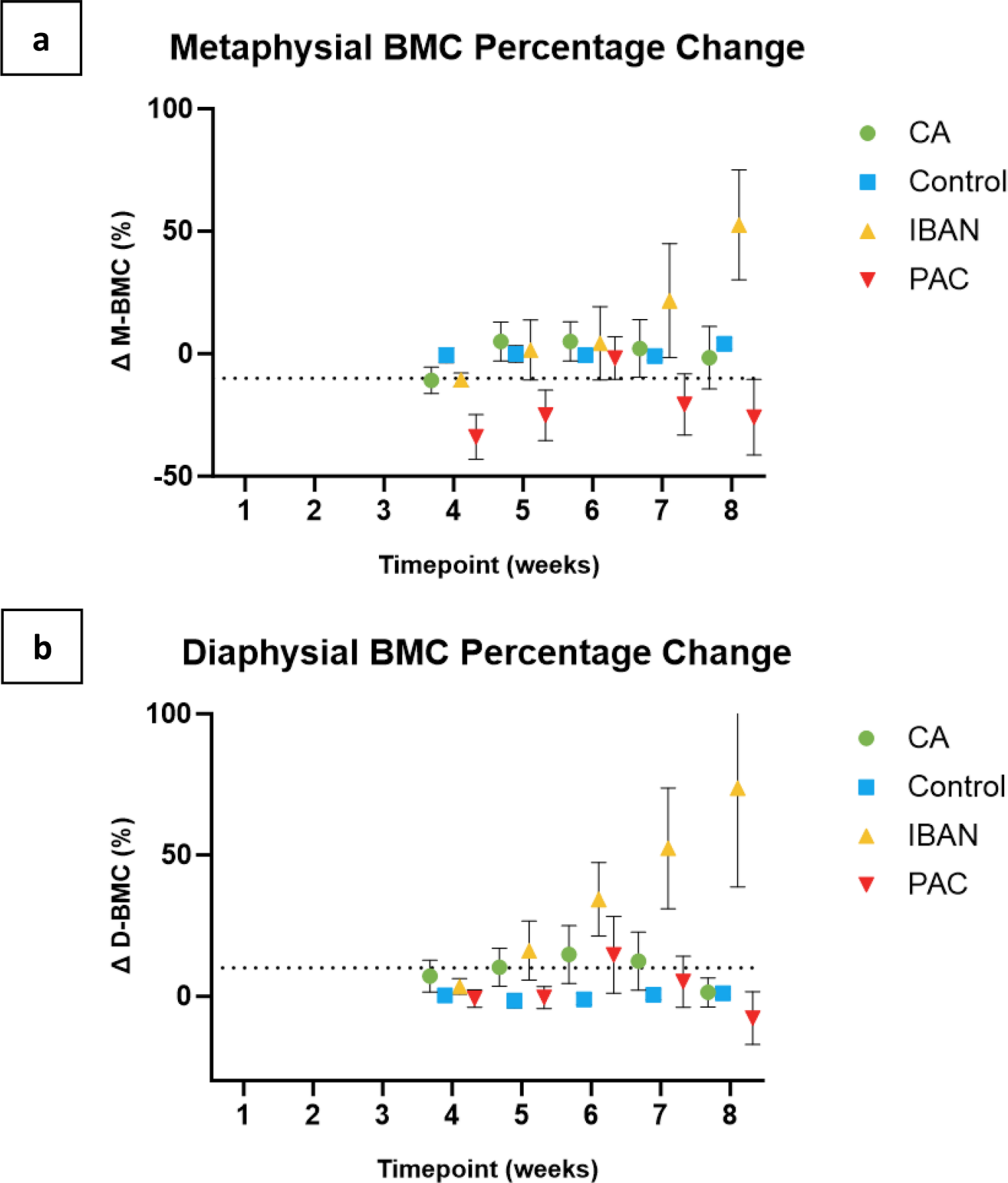
Demonstrating the weekly comparisons of the metaphysical (**a**) and diaphyseal (**b**) BMC. The dashed lines indicate the predetermined thresholds for fracture prediction

Metaphyseal BMC measured by DEXA yielded no significant differences between the groups (*p* > 0.05). Although diaphyseal BMC revealed a significant main effect of treatment (*p* = 0.015) and significant interaction between time point and treatment variables (*p* = 0.001), *post-hoc* pairwise comparisons did not reveal any significant differences between treatments at specific time points [Fig. 7a and b].

**Fig. 8:**
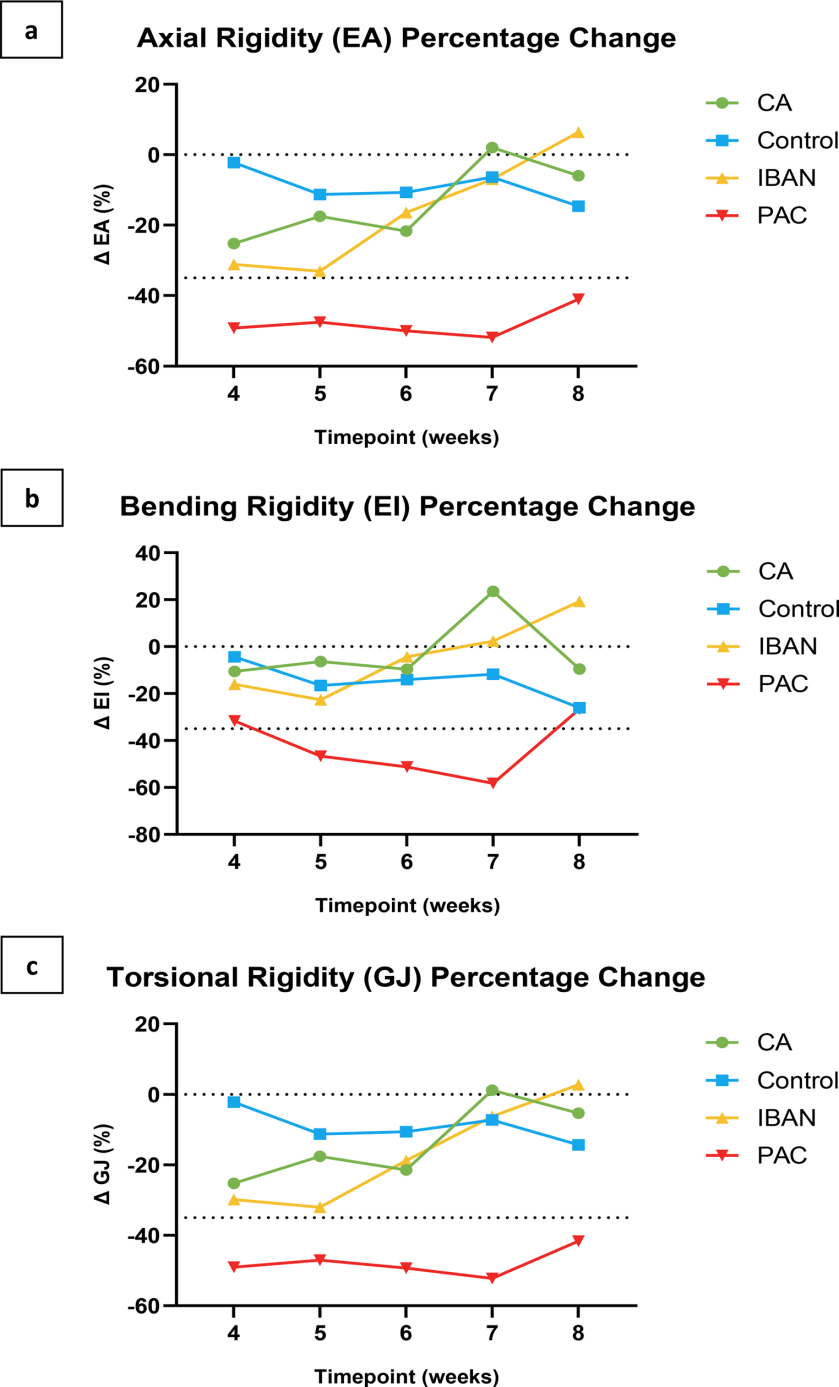
Demonstrating the trends of axial (**a**), bending (**b**), and torsional (**c**) rigidities between treatment groups. The dashed lines in the graphs denote either the baseline level, represented as zero, or the established threshold for fracture prediction

Axial, bending, and torsional rigidities of the affected limbs were normalized by the rigidity results of the homologous regions of the unaffected contralateral limbs. This provided a percentage change, where negative values indicate a loss of rigidity and positive values indicate a gain in rigidity from baseline, as a preferred mode of communicating results by orthopedic oncologists [Fig. 8a, b, and c].

**Fig. 9:**
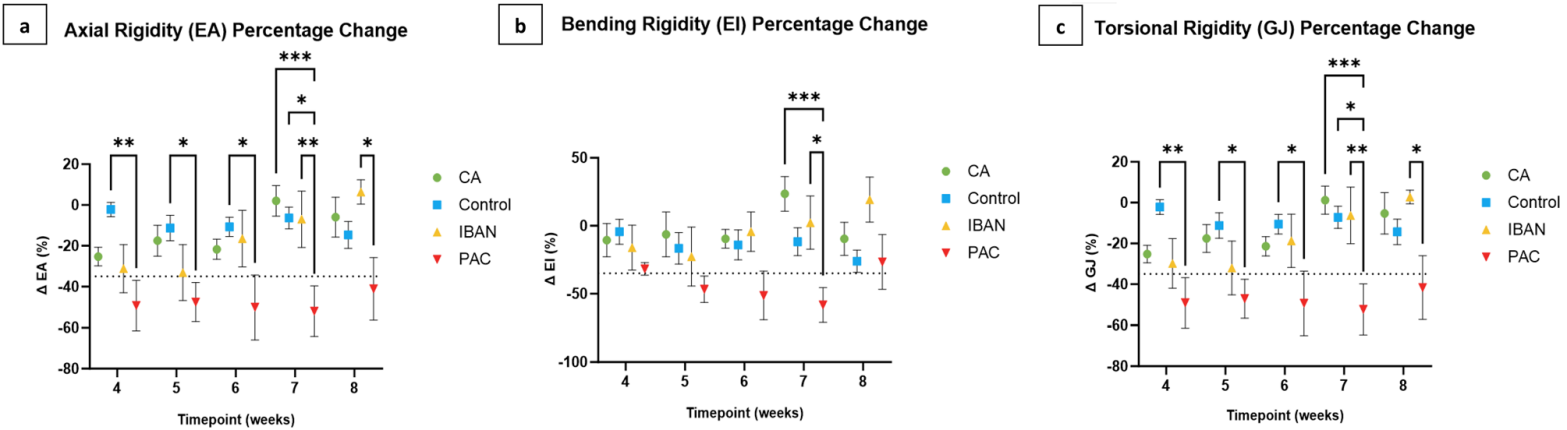
Demonstrating the weekly comparisons of axial (**a**), bending (**b**), and torsional (**c**) rigidities. * indicates P ≤ 0.05, ** indicates P ≤ 0.01, and *** indicates P ≤ 0.001. The dashed lines indicate the predetermined thresholds for fracture prediction

EA, EI, and GJ showed little variation across transaxial CT images as a function of time for the Control group, thereby establishing the stability of the technique to compare homologous slices and normalize rigidity measurements by the contralateral limb. Statistical analysis indicated a significant main effect of treatment on EA, EI, and GJ, demonstrating significant differences in the dependent variables across the treatment groups when considering all time points together. Post-hoc pairwise comparisons were conducted to investigate further the significant main effect of treatment at each time point. *Post-hoc* pairwise comparisons revealed notable differences between treatment groups for the EA, GJ, and EI outcomes [Fig. 9a, b, and c]. For the EA and GJ outcomes, the PAC treatment group showed significant differences compared to the Control group at weeks 4 to 7. Additionally, PAC was significantly different from the IBAN treatment and CA treatment groups at week 7. In the case of the EI outcome, *post-hoc* analysis demonstrated that the PAC group was significantly different from both CA and IBAN treatment groups, but only at week 7. These findings highlight the distinct impact of the PAC treatment on the EA, GJ, and EI outcomes compared to other treatment groups at specific time points.

## Discussion

The occurrence of skeletal diseases can lead to alterations in the quality (lamellar organization and mineralization) and quantity of bone through biological up-regulation or down-regulation of normal bone remodeling processes. If alterations in bone material and structure can serve as indicators of the interaction between metastatic lesions and bone, then the mechanical properties of bone could potentially be utilized for monitoring the effects of these modulators of tumor growth contributing to the extent of deterioration of the bone structure. In a series of *ex-vivo* [8, 36] and *in-vivo* [37, 38] experiments, our previous research has successfully demonstrated that the reduction in the Load-bearing capacity of bone with metastatic tumor can be predicted non-invasively. Employing QCT-based analysis, researchers were able to determine the Load-bearing capacity of vertebrae infiltrated with metastatic breast carcinoma. This study successfully predicted the occurrence of a new vertebral fracture with a sensitivity of 100% and a specificity of 72% [11]. These findings are markedly different from the best available fracture risk criteria, which are determined by the size and location of lesions on spine CT images and have only a 22% specificity [39, 40].

For this study, a fracture prediction threshold was established as a 33% reduction in any rigidity index compared to the contralateral femur, a score of more than 2 on the X-ray, or a 10% decrease in BMC compared to the contralateral femur. In our study, we utilized a threshold-based four-point categorical scale to assess radiographs, focusing solely on the lesion size. This approach is analogous to the size component of the Mirels’ criteria, adapted for use in our animal model. A critical value of 2 (lesion < 50% diameter of the bone) was chosen as the predictive fracture threshold. Any animal that scored equal to or greater than 3 (lesion size > 50% diameter by at least one observer) was predicted to fracture. Radiographic scoring occasionally demonstrated discrepancies between the two independent observers. Despite these inconsistencies, the inter-rater reliability, assessed using the kappa statistic, was significant, indicating a substantial agreement overall. Notably, there were instances, specifically concerning 4 animals, where one observer scored a lesion as indicative of a fracture (score of 3), while the other observer categorized the same lesion as non-fractured (score of 2). This discrepancy indicates the subjectivity and the inherent observer bias in X-ray visual scoring assessments. As a result, given the sensitivity to subjective interpretation and observer bias, relying simply on X-ray radiographs may not be the most accurate and reliable technique for evaluating certain conditions such as metastatic bone pathologies. Although the Mirels method of predicting fracture risk has been a valuable tool in the past, its methodology is vulnerable to the subjective interpretation of pain and lesion type [41, 42]. The Mirels method has been shown to have only 35% specificity with variations amongst different anatomical locations, leading to 2/3 of surgical cases undergoing unnecessary surgery and the associated complications [43]. In a study by Nazarian et al., they reported that the Mirels’ criteria were secondary to pain and lesion type in the clinical decision-making process [41].

On the other hand, BMC, as an objective measure, could differentiate the overall difference between the groups, but the *post-hoc* analysis for this modality was non-significant. This demonstrates that while the treatment effect is more apparent when assessing the cumulative effect across all time points, BMC fails to distinguish between these treatments when examining differences at particular time points. This study shows that CT-based structural rigidity analysis captures progressive changes in bone structure over time that represent tumor progression or response to treatment better than biplanar radiographs or DEXA. Furthermore, CT-based rigidity analysis provided more nuanced insights into potential fracture risks than radiographic analysis based on lesion size or DEXA measurement of bone mass. For example, rat OP44 from the PAC group exhibited a significant decrease in EA, EI, and GJ at the lesion site four weeks post-inoculation, exceeding 33% from the baseline. The results of the study revealed that there was a gradual decline in rigidity for this animal from the fourth to the seventh week, as indicated by EI, which was in agreement with the BMC findings; however, the X-ray analysis only detected a fracture in the fourth week, and the observers did not report any fractures in the following weeks. In another observation, rat OP56 from the IBAN group was predicted to have fractures based on all rigidity indices from week 4 to the end of observation at week 8. This was consistent with the BMC threshold, but X-ray imaging only detected the fracture at week 8. These observations suggest that CT-based rigidity analysis might offer earlier indications of structural vulnerabilities that could lead to fractures, though these predictions should be interpreted with caution due to the lack of direct fracture verification in the study.

Furthermore, after fracture prediction, several rats demonstrated robust fracture healing with extensive callus formation and concomitant periosteal expansion that partly compensated for the mechanical effect of the osteolytic lesion. In a similar observation within the IBAN group, rat OP53 was identified as having fractures on all three modalities at week 4. Subsequently, while CTRA continued to indicate a fracture risk at week 5, X-ray assessments showed perceived fractures up until week 7, only to classify the femur as non-fractured by week 8. These findings suggest that CTRA may offer a more sensitive and objective method for detecting dynamic changes in bone integrity than conventional biplanar radiography.

Bisphosphonates have the ability to suppress resorption via osteoclasts, decelerate the advancement of bone metastases, and might prevent extraosseous metastases by inducing apoptosis and obstructing the metastasis cascade along with the subsequent angiogenesis [44]. Ibandronate is a member of the bisphosphonate class of drugs and was the exclusive bisphosphonate applied in our research. It stands out as an extremely effective agent in managing and averting hypercalcemia, pathological fractures, and bone pain in patients suffering from metastatic BrCa. The inter-animal response to bisphosphonate treatment (IBAN) varied widely. A number of animals showed slight improvement, others experienced substantial enhancements in stiffness, while some showed no progress. This variability underlines the necessity to formulate a method that monitors an individual’s reaction to both the tumor and treatment over time. Relying solely on assessments of fracture risk based on the dimensions and position of an osteolytic lesion is inadequate since these analyses overlook the structural properties of the host bone that might be influenced by concurrent conditions like osteoporosis, or they fail to consider the compensatory effect of the tumor-induced bone formation neighboring the lesion. The observations in the IBAN group, which indicate an increase in rigidity over time, prompt a valuable discussion regarding the impact of antiresorptive treatments on bone architecture. Ibandronate, by inhibiting osteoclastic activity, may preserve or even enhance the bone mineral content, particularly within the trabecular bone compartment [45]. The preservation of trabecular integrity could contribute to the increased rigidity measured by qCT, as trabecular bone plays a critical role in resisting compressive forces. The enhanced rigidity despite the presence of osteolytic lesions can be seen as an effect of the drug’s action on the cancellous matrix, which may not be directly paralleled by changes in cortical bone. This underlines the importance of considering compartment-specific responses when evaluating the effects of antiresorptive therapy on bone health. It also highlights the nuanced relationship between changes in BMC and structural rigidity, suggesting that increases in rigidity in the IBAN group may not solely reflect the status of lesion progression but also the pharmacological influence on bone turnover dynamics [46].

The authors acknowledge several limitations within the study. Firstly, the use of pQCT limited our ability to accurately measure tumor volume progression due to its lower resolution compared to microCT, which could potentially affect the detailed analysis of lesion progression over time. Additionally, the study’s reliance on the contralateral limb as a control in systemic treatment scenarios could introduce biases if the systemic effects alter bone mineral content uniformly, which was not accounted for in our comparative analysis. Moreover, the sample size may have been too small to detect statistically significant differences in some of the observed changes in bone rigidity, which could influence the robustness of our findings. Furthermore, the absence of histological analysis limits the direct correlation of CTRA findings with actual biological changes within the bone, which could impact the study’s validity in predicting fracture risks based on bone rigidity changes. Additionally, the study did not include direct post-mortem fracture verification which poses a limitation, as it impedes our ability to correlate CTRA predictions with actual fracture events. Lastly, while the study provides valuable insights into the use of CTRA in a preclinical setting, the direct applicability of these findings to human clinical scenarios requires further investigation to account for interspecies differences in bone biology and disease progression.

The local bone structure accurately reflects the interactions between host bone and metastatic cancer, symbolizing a dynamic battlefield where both the host and invasive cancer cells implement strategies for survival and proliferation. Bone, being a dynamic tissue, is incessantly undergoing remodeling, a process orchestrated by osteoblasts and osteoclasts. Interactions at the continuum level are overseen by meticulous governance due to factors released by the tumor and other systemic regulators of cellular activities. These interactions result in modifications in the structural and material properties of the bone, altering its cross-sectional geometry and intrinsically changing its mechanical properties. The structural properties, identified through advanced imaging modalities, offer insights necessary for tracking the impact of the biology of metastatic cancer on the host bone, assisting in the prognosis and management of metastatic bone diseases. By understanding the clinical implications of the interaction between metastatic cancer and local bone structure, clinicians can customize therapeutic interventions to counterbalance the harmful alterations induced by the metastatic cells, potentially enhancing the quality of life for patients with metastatic bone disease.

## Conclusion

The findings of this study further validate that the gap between clinical guidelines and physician’s recommendations in the decision-making process for selecting surgical or non-surgical treatment must be narrowed by more advanced prognostic tools such as CTRA. In this study, CTRA has indeed demonstrated its capability to assess the progression of osteolytic lesions, as well as the tracking response to therapeutic interventions within our rat model of metastatic bone lesions. Contrary to existing methodologies, CTRA has provided a more comprehensive insight into tumor- and treatment-induced spatial and temporal alterations in bone mechanics. Our ultimate goal is to expand this study to include additional treatment options and larger group sizes to evaluate the value of CTRA in a large animal cohort exposed to a host of clinically relevant treatment options over time.

## Data Availability

The datasets used and/or analyzed during the current study are available from the corresponding author on reasonable request.

## Abbreviations

ANOVA: Analysis of Variance
BrCa: Breast Cancer
BMC: Bone Mineral Content
CDST: Clinical Decision Support Tool
CTRA: CT-based Structural Rigidity Analysis
DEXA: Dual-Energy X-ray Absorptiometry
EA: Axial Rigidity
EI: Bending Rigidity
GJ: Torsional Rigidity
QCT: Quantitative Computed Tomography
LBC: Load-bearing capacity
